# Effects of prematurity and socio‐economic status on early life language exposure: A video coding study

**DOI:** 10.1111/bjdp.70023

**Published:** 2025-11-24

**Authors:** Sinéad O'Carroll, Bethan Dean, Yu Wei Chua, Victoria Ledsham, James P. Boardman, Sue Fletcher‐Watson

**Affiliations:** ^1^ Salvesen Mindroom Research Centre University of Edinburgh Edinburgh UK; ^2^ Strathclyde Institute of Education University of Strathclyde Glasgow UK; ^3^ Centre for Reproductive Health, Institute for Regeneration and Repair University of Edinburgh, the Queen's Medical Research Institute Edinburgh UK

**Keywords:** deprivation, gesture, language, parenting, preterm birth, socio‐economic status, speech

## Abstract

Preterm birth is associated with later language impairment and delay. Socio‐economic deprivation is linked to decreased language exposure in early childhood, but it is unknown how prematurity influences this relationship. This study investigated the effects of socio‐economic status and gestational age at birth on language exposure, in a sample of 47 preterm and 53 term infant‐parent dyads. Videos were coded for parental language and gesture using a novel coding scheme. Video codes revealed no significant differences in the language and gesture used by parents of preterm infants versus term infants during interaction. Socio‐economic status was positively correlated with quantity and variety of words used, and mean length of utterance (all *r* > .25, *p* < .015). Deprivation, and especially the increased prevalence of deprivation among the preterm population, may be a key factor to consider when examining the risk of language delay for infants born early.

**Statement of Contribution**

**What is already known on this subject?**

- Preterm birth is associated with language impairments and delay.
- Socio‐economic deprivation is associated with reduced language exposure in early life, and is also over‐represented among infants born early.

**What the present study adds?**

- Preterm infants as a group do not receive less quantity or reduced quality of language exposure from their primary caregiver compared with infants born at term.
- Deprivation is associated with reductions in amount and richness of speech but not in gesture.

## INTRODUCTION

Preterm birth is defined as any baby born alive before 37 weeks of pregnancy. Preterm birth is associated with an increased likelihood of difficulty in many neurodevelopmental domains, including a higher risk of later language impairment and delay (Sansavini et al., 2010; Valavani et al., 2022; van Noort‐van der Spek et al., 2012). This pattern is also found in sub‐clinical measures of language outcome. For example, 2‐year‐old preterm children were found to have a similar vocabulary score range as term‐born peers, but the preterm population was overrepresented at the lower end of the range and lower vocabulary was associated with lower gestational age at birth (Foster‐Cohen et al., 2007). Another study found a normative lag of approximately 3 months in age‐equivalent communicative ability among preterm children (Cattani et al., 2010).

There is mixed evidence regarding the pre‐linguistic behaviours of infants born prematurely. Canonical babbling and rhythmic hand banging were found to be at an earlier onset in preterm versus term‐born infants, which would suggest an advantage for later language learning (Eilers et al., 1993). A later study found that preterm infants did not exhibit a delay, but did produce fewer more advanced vocalizations, and showed a delay in first word production (Törölä et al., 2012). One possible reason for this pattern is that canonical babbling in the preterm population may not be as stable as it is in the term‐born population (Oller et al., 1994). Turning to gesture, gesture production predates speech and can be used to predict later language production (Bates, 2014; Iverson & Goldin‐Meadow, 2005). Benassi and colleagues found a delay in early gesture production in very early preterms, and proposed that this early disruption may have a negative impact on later language development (Benassi et al., 2016). De Schuymer and colleagues observed delays in pointing and gaze‐following, as well as later delays in expressive and receptive language, when compared with infants born full term (De Schuymer et al., 2011). Preterm children have also been found to produce fewer gesture and word combinations at 18 and 24 months compared to children born full term, suggesting that gesture production may be a key aspect for understanding the communicative skills of preterm infants (Suttora & Salerni, 2011).

Candidate mechanisms driving the delayed or otherwise atypical language development of infants born preterm include: the impact of factors that are prevalent among parents who deliver early, such as socio‐economic deprivation (McHale et al., 2022; Nguyen et al., 2019) or drug use (Baer et al., 2019); the impact of early birth on brain development underpinning language skills (Stipdonk et al., 2018); and the impact of early birth on the parent–child relationship (Ionio et al., 2017). However, little is known about the relative contribution of these various factors. The current study aims to provide insights that will support efforts to distinguish between some of these potential explanations. In doing so, our focus is on parental language input in the early stages of infant communication development. This can help reveal sensitive periods and targets for early language interventions, with the goal of instigating substantial downstream benefits in childhood and beyond (Friedmann & Rusou, 2015).

Research has found significant evidence for a robust, and strong relationship between parent language input and infant language development (Iverson et al., 1994; Schmidt & Lawson, 2002; Zammit & Schafer, 2011). The quantity, richness and complexity of maternal speech have each been found to benefit the lexical development of young children (Hoff & Naigles, 2002) and the volume of word types in maternal speech is related both to children's receptive and expressive vocabularies (Bornstein et al., 1998). Infants born preterm are exposed to less maternal voice and increased levels of noise due to their experience in the neonatal intensive care unit, or NICU. Exposure to parental vocalizations is a strong predictor of infant vocalizations at 32 weeks and conversational turns at 32 and 36 weeks, compared to language input from other adults (Caskey et al., 2011). Higher adult word count in the NICU is also associated with higher infant language scores at 7 months and higher expressive communication scores at 18 months, suggesting that early language input can have long‐lasting implications (Caskey et al., 2014). There is evidence that parents who experience preterm labour and spend weeks with their infant in intensive hospital care can be profoundly affected by the experience, in ways that have a lasting impact on their parenting (Treyvaud et al., 2014). However, variability in parent language after the NICU stay has ended is less easy to observe and therefore less well understood.

Another aspect of the infant's language exposure is parental use of gesture. Children's language learning is facilitated through interactions with adults and the establishment of early labelling and language ‘routines’ (Acredolo & Goodwyn, 1988). Parents use a combination of gesture and speech to ‘*encourage children to orient to aspects of the environment’* (Schmidt & Lawson, 2002, p.3), and parental gesture helps to foster children's joint attention skills and is predictive of later language outcomes (ibid.). Maternal pointing positively correlates with children's later vocabulary size (Iverson et al., 1994) and more maternal gesturing is associated with increased word learning in children (Zammit & Schafer, 2011). Increased levels of joint engagement between mothers and children are associated with larger comprehension vocabularies (Carpenter et al., 1998). Little is known about how parental gesture varies for parents of infants born preterm, though as previously noted, preterm infants' own gestures are related to their language outcomes.

Another factor that may further contribute to the association of prematurity and poor language outcomes is deprivation. There is an increased risk of language delay unequally experienced by infants from households with a low socio‐economic status (Huttenlocher et al., 2010; Pungello et al., 2009). In a hallmark study, 3‐year‐old children from a high socio‐economic status household were found to have double the number of words in their vocabularies as children with a low socio‐economic status (Hart & Risley, 1995). Such children experience delays specifically in regard to vocabulary development, and rates of gesture (Fernald et al., 2013; Pungello et al., 2009). This difference is apparent as early as 18 months and continues to be significant at 24 months (Fernald et al., 2013). Additionally, children from low socio‐economic status homes exhibit slower rates of vocabulary growth (Blanden & Machin, 2010). Studies have reported a positive correlation between high socio‐economic status and increased vocabulary development (Arriaga et al., 1998). Early gestural differences have also been observed, with children from low socio‐economic status families producing less gesture to communicate meaning (Rowe & Goldin‐Meadow, 2009).

The over‐representation of children from a deprived background on measures of language delay is thought to result, in part, from a lack of exposure to rich and complex parental language (Bradley et al., 2001; Hoff, 2003; Vernon‐Feagans & Bratsch‐Hines, 2013). Children from a low socio‐economic status background are exposed to less language than children from high socio‐economic status homes (Hart & Risley, 1995). Mothers with a low socio‐economic status may use shorter mean length utterances than mothers from high socio‐economic status backgrounds (Hoff, 2003). This is consequential as the children in this study who were exposed to longer utterances displayed a faster rate of vocabulary growth (Hoff, 2003). Lexical complexity and diversity in maternal speech have also been found to relate to socio‐economic status, and exposure to child‐directed speech has been found to act as a buffer in children from low socio‐economic status who are at a higher risk for poor language outcomes (Huttenlocher et al., 2010; Vernon‐Feagans & Bratsch‐Hines, 2013). Rowe found that mothers with a higher level of education had a higher level of pointing, specifically using their pointing gesture to direct conversations and focus attention (Rowe, 2000). This builds upon previous research suggesting that pointing can help to establish joint attention between parents and children, which itself is important for language development (Iverson et al., 1994).

Mothers from a deprived neighbourhood are also at a significantly higher risk of preterm delivery, and the preterm population is over‐represented on the lower end of the socio‐economic spectrum (Bonet et al., 2013; Mehra et al., 2019; Peacock et al., 1995). This often results in an imbalance of representation of socio‐economic status in studies of prematurity (Cusson, 2003). Consequently, in studies that do not control for or consider socio‐economic status, any resulting language differences in term‐born and preterm children may not be a true reflection of a prematurity risk, but may in fact be a confounding and unmeasured risk of deprivation. The literature has further shown that socio‐economic advantage can have a protective effect over infants who are otherwise at an increased risk of adverse outcomes (Kurstjens & Wolke, 2001; Stein et al., 2008) including infants born preterm (Ene et al., 2019). One cohort study found that higher socio‐economic status, here operationalized as maternal education, helped to mitigate the developmental impact of brain injury within a group of preterm neonates (Benavente‐Fernández et al., 2019). Low socio‐economic status and prematurity can often be thought of as ‘separate risk factors with multiplicative effects on developmental delay’ (Potijk et al., 2013, p.26).

It is clear that there is a complex but robust relationship between prematurity, socio‐economic deprivation and language outcome. While we do not fully understand the mechanisms through which these relationships operate, it is evident that some preterm infants and children from more deprived homes are at increased risk of language delay or disruption. From these established relationships, it follows that there is a low socio‐economic status, preterm sub‐group that emerges as being at a significantly increased risk of adverse language development. One theoretical foundation for this is the Family Stress Model (Masarik & Conger, 2017), which articulates how family hardship may impact child development, mediated by factors such as disrupted parenting. In this case, the combined stress of socio‐economic deprivation and raising an infant born prematurely is posited to impact parental communication and thus infant language development.

Examining parent communication in the early years through this theoretical lens, may reveal one of the mechanisms by which this risk takes effect, as well as indicators of potential intervention targets. Additional insight can be generated by capturing multiple metrics of deprivation. Family‐level measures may operate more dominantly in very early life, while neighbourhood deprivation could become more influential across childhood (McKinnon et al., 2023), also influencing quality of life (Masarik & Conger, 2017). Meanwhile, although maternal educational level is often used as a measurement of socio‐economic status, it is also found to have a direct influence on the language mothers use for communication (Braveman et al., 2005; Tracey & Young, 2002). Thus, for the purposes of this study, we capture both maternal education and neighbourhood deprivation, as well as quality of life, as candidate influencers on infant language exposure.

This study takes place during the first year of life, when infants are pre‐verbal. We have already established the role that child gesture plays in their language development.

To that end, our research questions are:

1. Do parents of infants born preterm show different patterns of speech and gesture use with their infants, compared with parents of term‐born infants?
2. Do factors related to socio‐economic status (neighbourhood deprivation, maternal education and quality of life) contribute to variability in parental speech and gesture use, within or across preterm and term‐born groups?

## METHOD

### Participants

Participants were recruited as part of the Theirworld Edinburgh Birth Cohort, Phase 2 (Boardman et al., 2020). Data were collected from a total of 122 infants. Of this sample, 22 infants were excluded from the final analysis. Seventeen infants were excluded as their parent–child play involved a language other than English, while two were excluded from analysis due to a technical error involving the video camera. One infant was excluded as they were called back for a research visit too early and thus fell outside of the included age range. Two infants were excluded from analysis as their videos were used for the training of the second coder.

Data were analysed from 100 participants in total. The preterm group consisted of 47 infants born <32 weeks of gestation (mean gestational age of 29 weeks, range of 24–31 weeks). The term‐born comparator group consisted of 53 infants born >37 weeks of gestation (mean gestational age of 39 weeks, range of 36–42 weeks). Gestational age differed significantly between groups (*t*(98) = 34.9, *p* < .001), with a mean difference of 10.15 weeks. Birthweight also differed significantly between groups (*t*(98) = 26.8, *p* < .001), with a mean difference of 2241 g. There were no significant mean differences between preterm and term‐born infants on SIMD, or Scottish Index of Multiple Deprivations, rank, (*t*(90) = 1.112, *p* = .269), maternal level of education (*t*(90) = 1.415, *p* = .161) or WHOQOL‐BREF scores (*t*(86) = 1.400, *p* = .165) which is a quality of life measure (World Health Organization, Quality of Life – Brief). Group demographics are shown in Table 1. Parents who took part were all primary caregivers and included a small minority of fathers (*n* < 5); due to the very small sample and risk of identification, we do not distinguish further based on parent gender.

**TABLE 1:** Participant demographics by group.

Variables	Preterm	Term
	*n* = 43	*n* = 49
Gender (m:f)	30:17	28:25
Clinical comorbidities		
Sepsis	5	0
Necrotizing Enterocolitis (NEC)	1	0
Bronchopulmonary Dysplasia (BPD)	7	0
Mean (SD) [Range]		
SIMD Rank	4180 (2036) [137–6929]	4638 (1914) [727–6936]
Maternal Level of Education	4.49 (1.40) [1–6]	4.88 (1.24) [1–6]
WHOQOL‐Bref Raw Score	8.18 (1.29) [5–10]	8.60 (1.44) [4–10]

Infants with congenital anomalies, here defined as structural or functional anomalies (e.g. metabolic disorders) that occur during intrauterine life and can be identified prenatally, at birth or later in life, were excluded from the study. These include anomalies such as heart defects, neural tube defects and Down syndrome. Infants with a contraindication to MRI at 3 Tesla, such as those with an implanted medical device were also excluded. This was done as these congenital anomalies are known to be associated with poor developmental outcomes and consequently it would have been difficult to separate delays due to congenital anomalies from outcomes due to prematurity.

### Procedure

Parent–infant dyads were assessed at 9 months corrected age (range of 8–10 months) for the preterm group and 9 months chronological age (range of 8–11 months) for the controls. Parent and infant dyads were filmed during a 10‐min free play protocol that occurred in a small testing room in a University building. During parent–child play, infants and parents were presented with a series of toys: a small plastic car, a sound‐producing ball, blocks, a doll with a removable hat, a blanket and bottle and a fabric book. All parent and infant pairs had access to the same set of toys, which are a similar selection, in terms of their affordances, to those used in other infant studies (see Iverson et al. 2008, Goldin‐Meadow et al., 2007). Parents were instructed to play with their infants as they would normally do at home. In cases of bilingual or multilingual speakers, parents were advised to interact in the language that they would typically use in a home environment. Only samples that contained fewer than 10 instances of a language other than English were included in this study, and, as noted above, 17 samples were excluded for this reason. In the large majority of these cases, the instances were of Scots dialect in families who did not report bilingualism, but whose use of Scots nonetheless impeded the coder's understanding. Researchers left the room during the parent–child play.

### Measures

Video coding was chosen as an appropriate method to capture naturalistic gestures and speech of parents when interacting with their infants (Fusaro et al., 2014). Parents were unaware of the types of behaviours being coded, thereby minimizing the impact of the observational method on their behaviour.

Following previous work, all parental speech was coded, even when not identified as addressing the infants directly, as non‐directed language was still serving as a language stimulus (Sung et al., 2013). Non‐syllabic noises such as hissing or shushing were excluded, while transliterated sounds and vocalized exhales, for example, ‘Ah’ or ‘Ah ba ba’ were included. Additionally, we did not code laughter, or mouth and lip manipulations that are not vocalized, such as blowing. Finally, we did not include coughing or vocalized inhales in our coding, as coughing is involuntary and vocalized inhales are too inconsistent to be considered a reliably coded variable. Using protocols typically followed in the case of code switching bilingual French/English speakers, any Scots words or common Scottish colloquialisms were coded as word types (Genesee et al., 1996). As per Genesee, Nicoladis and Paradis, utterances that deviated from typical pronunciation were coded phonetically (Genesee et al., 1995). A standardized list of commonly used Scots words and colloquialisms was provided to the second coder, to ensure standardized spelling across codes.

Videos were first transcribed for parental speech, noting each time‐stamped utterance. Speech was divided into utterances, whereby utterances are divided by a change in conversational turn, a change in intonation or a silence for at least 10 ms (Iverson et al., 1999). Utterances unidentifiable as words were coded phonetically. Similarly, any parts of an utterance that deviated from typical pronunciation were coded phonetically (Genesee et al., 1995). Once coding of language was complete, the transcription of each video was exported into a CHAT delimited text file. Transcriptions were then analysed by human coders using CLAN (Computerized Language Analysis) (MacWhinney, 2018).

Following this, videos were then coded for parent gesture. A novel coding scheme was created to capture parental movements when communicating with their preverbal infants. Each movement made by the parent was coded for duration of movement, handedness and type of movement. The parameters of each gesture were defined by either a pause in the movement or an obvious change in the shape or trajectory of the movement (see Kong et al., 2016). Gestures were coded globally, meaning that the whole gesture was coded as a unit, rather than separating the individual flexion and extensions of the hand or arm. For the purpose of coding, gestures can be thought of similarly to vocal utterances, as they are also defined by moments of physical rest or a change in intention. Gesture categories included manipulating object, manipulating infant, pointing, giving and other. This inclusive set of categories was developed after initial pilot testing which revealed that a majority of gestures involved physical contact with the infant or an object. While previous research largely reveals the influence of canonical gestures such as pointing on language development, we wanted to capture all potentially relevant communicative gestures from the available sample. For example, in this age range, we found that parents may physically orient an infant so that a specific item is in their view and while this is not a typical gesture, since it involves physical contact, it does have clear communicative intent. Manipulations of the infant could also signal emotional meaning, as in an embrace or stroke of the hair, and thus augment speech with additional communicative content. See Table 2. for a comprehensive definition of what was included in each gesture category.

**TABLE 2:** Gesture code descriptions.

Code	Description
Giving	Defined as any instance of the parent holding an object out with the intention of giving it to the infant. This also included instances of the parent holding out hands/presenting them to the infant to take. Rolling a ball towards an infant was not considered as giving but was instead classified as a manipulation of object. In instances where a parent was holding an object and the infant took it from their hands, with or without any obvious parental intention of directly giving, this was also coded as giving
Manipulating infant	Defined as any instance where a parent physically interacts directly with the infant. This includes forms of touching without movement of the infant, such as putting a hand on the infant's back for support or brushing an infant's hair. Any physical limitations of infants, such as needing extra support to sit upright, were noted in the case of potential outliers. When parents would lift or carry infants, this was also coded as a manipulation of the infant, with the code being separated by any change in movement or intention. For example, a code would start when a parent lifted the infant in the air, and end when the movement changed to pulling them closer to their body
Manipulating object	Defined as any instance of the parent moving or manipulating an object. This code included any time the parent touched a toy or the play mat. In instances where the parent touched a personal item, such as a tissue or pacifier these were coded as manipulating object but it was noted that the object was external
Pointing	Defined as any gesture used to indicate towards a particular object/location of interest through the use of a single finger extended outwards. While pointing gestures are typically defined as an extension of the index finger, given that parents of 9 month olds are often holding other things during interactions, this has been expanded to include any single finger extension. Initial viewings of the video indicated that this was a necessary expansion, as parents will often use their thumbs to point if their index finger is occupied with another object. For example, when holding the ball or book, parents will often use their thumb to point while their index finger is involved in the manipulation or holding of the object. This is similar to the parameters of the pointing gesture as defined by Iverson et al. (1999)
Other	Defined as any gesture that does not fit into the classification codes detailed previously. This could include instances of classic iconic gesture, such as manipulating fingers to indicate a heart shape or separating hands with palms facing inwards to indicate the size of something. Clapping, waving and classic beat gestures, the random hand and arm movements made when speaking to add emphasis onto certain words, were also coded as other. Every gesture coded as other was briefly described directly in ELAN

A second coder, blind to the infant group, was trained by the primary coder, and then independently rated a random selection of 10% of the videos, to ensure inter‐rater reliability. The agreement rate between coders for the total number of gestural events was 94%, and Cohen's kappa was .745 for the classification of gestures. Intra‐class correlations to measure agreement between coders on language measures were an average of 98% (range 0.983–0.998 ICC). These indicate excellent agreement across all codes. See Table 3. for a comprehensive list of what was and was not included in the coding process.

**TABLE 3:** Inclusion and exclusion criteria for coding scheme.

Coded variables	Included	Excluded
Language	Words	Non‐syllabic noises
	Utterances identifiable as words	Laughter
	Parental babbling	Mouth and lip manipulations
	Transliterated sounds	Coughing
	Vocalized exhales	Vocalized inhales
Gesture	Manipulating object	Parent touches own face
	Manipulating infant	Parent adjusts own clothing
	Pointing	Parent shifts own body
	Giving	Parent stretches
	Other	

Socio‐economic status (SES) was measured using the Scottish Index of Multiple Deprivation 2016, or SIMD 2016 (Scottish Government, 2016). The SIMD 2016 is a multilevel tool developed and used by the Scottish government to capture area‐level deprivation (Scottish Government, 2016). The SIMD 2016 splits Scotland into 6, 976 data zones all with similar population sizes. These data zones are then examined for multiple indicators of deprivation, including factors such as travel times to GP, school pupil attendance and unemployment rates. These indicators of deprivation are grouped into seven domains (employment, income, crime, housing, health, education and access), which are then grouped into one SIMD. This results in each data zone in Scotland being ranked from 1 (most deprived) to 6976 (least deprived), (Scottish Government, 2016). SIMD scores were therefore extracted from the Scottish Government rankings, based on the participants' postal code.

Parents completed the self‐report WHOQOL‐BREF questionnaire as part of a questionnaire pack provided prior to the appointment. The WHOQOL‐BREF is an abbreviated version of the WHOQOL‐100 quality of life assessment (Webster et al., 2010) with good discriminant validity, content validity, internal consistency and test–retest reliability (WHOQOL Group, 1998). The measure asks respondents to consider their ‘quality of life, health or other areas of your life’ over the preceding 2 weeks. Items capture physical, psychological, social and environmental aspects of quality of life.

Final level of maternal education was collected via a questionnaire directly administered to the parents by a research nurse during the perinatal period and interpretable within the education systems of Scotland, England, Wales and Northern Ireland. Options included: no education, and qualifications obtained at basic high school, advanced high school, college, university undergraduate, university postgraduate and the post‐natal appointment.

## ANALYSIS METHODS

A sensitivity power analysis (G*Power, Faul et al., 2007) showed that our sample was sufficient to detect moderate effect sizes (*d* = 0.5) at alpha .1 with a power of .8. The independent variables were preterm or term‐born status and markers of prematurity including birth weight, and gestational age. This helped to account for the wide range of gestational ages represented in the preterm group (24–31 weeks) which may have impacted parent behaviour. Analyses focused on relations between language and socio‐economic variables combined the preterm and term‐born groups into a single sample. Dependent variables were parental gesture and speech measures. Specifically, these were gesture rates in each gesture category (total number of gestures, manipulating object, manipulating infant, pointing, giving and other), number of word types, number of word tokens, type/token ratio and mean length of utterance. See Table 4 for a list of variables included in the analysis.

**TABLE 4:** Variables included in analysis.

Domain	Variables	Description
Prematurity	Birthweight	Birthweight, in grams, of infant measured at birth
	Gestational age	Gestation of pregnancy, measured in weeks, at birth
Language	MLU	Average length of utterance
	Word types	Total number of different words spoken
	Word tokens	Total number of words used during the interaction
	Type/token ratio	Number of word types divided by the number of word tokens
Gesture	Manipulating object	Any instance of the parent moving or manipulating an object
	Manipulating infant	Any instance where a parent physically interacts directly with the infant
	Giving	Any instance of the parent holding an object out with the intention of giving it to the infant
	Pointing	Any gesture used to indicate towards a particular object/location of interest through the use of a single finger extended outwards
	Other	Any gesture that does not fit into the classification codes detailed previously

We used t‐tests for comparisons between the two groups (preterm, term‐born) on each independent and dependent variable. We then conducted Pearson correlations to examine relations between independent and dependent variables. Finally, we conducted hierarchical linear regression to examine the explanatory power of candidate SES measures as predictors of parental communication.

## RESULTS

### Prematurity and parental language measures

Parents of term‐born and parents of preterm infants produced similar quantities of language and gesture when interacting with their infant during a parent–child play session, see Tables 5, 6.

**TABLE 5:** Language variables in preterm/term groups.

Codes	Term			Preterm		
	M	SD	SE	M	SD	SE
Word types	190.94	55.30	7.60	190.96	52.96	7.72
Word tokens	572.72	201.09	27.62	559.09	214.44	31.28
Type/Token ratio	0.348	0.06	0.01	0.36	0.06	0.01
Utterances	170.68	50.88	6.99	169.96	55.19	8.05
Mean length of utterance	3.313	0.55	0.08	3.26	0.54	0.08

*Note*: No significant differences at *p* < .05.

**TABLE 6:** Gesture variables in preterm/term groups.

Codes	Term			Preterm		
	M	SD	SE	M	SD	SE
Manipulating object	146.64	44.38	6.10	151.72	38.94	5.68
Manipulating infant	22.85	19.68	2.70	21.81	17.06	2.49
Pointing	10.09	6.82	0.94	10.06	9.55	1.39
Giving	7.92	5.31	0.73	8.83	5.86	0.86
Other	6.08	6.89	0.95	4.30	4.54	0.66
Total gesture	193.58	45.28	6.22	197.32	45.54	6.64

There were no significant differences in gesture frequency between groups. Frequency of manipulating infant (*t*(98) = .281, *p* = .779), manipulating object (*t*(98) = −.605, *p* = .546), pointing (*t*(98) =.019, *p* = .985), giving (*t*(98) = −.811, *p* = .419) and other gestures (*t*(98) = 1.503, *p* = .136) was all similar between preterm and term‐born groups. Additionally, there was no significant difference in total gesture frequency between groups (*t*(98) = −.411, *p =* .682). In terms of language, total number of word types (*t*(98) = −.001, *p =* .999), word tokens (*t*(98) = .328, *p =* .744), type/token ratio (*t*(98) = −.980, *p =* .330), total number of utterances (*t*(98) = .068, *p =* .946) and MLU (*t*(98) = .487, *p =* .627) were not significantly different between parents of term‐born and parents of preterm infants.

### Socio‐economic status, quality of life, and parental language measures

Given no effect of preterm birth on any parental language measure, data from term‐born and preterm infants were combined to investigate influences from socio‐economic status. SIMD was significantly positively correlated with word types (r = .258, *p* = .013), word tokens (r = .321, *p* = .002) and MLU (r = .373, *p* = .000). SIMD also showed a negative correlation with type/token ratio (r = −3.04, *p* = .003). Maternal education was significantly positively correlated with MLU (r = .285, *p* = .006) but no other communication variables. Finally, WHOQOL‐BREF was significantly positively correlated with manipulating infant (r = .241, *p* = .023), word types (r = .231, *p* = .030), word tokens (r = .274, *p* = .010) and MLU (r = .230, *p* = .031). See Table 7 for the full results of these analyses.

**TABLE 7:** Correlation matrix.

Variables	SIMD rank	Maternal level of education	WHOQOL
*N*	92	92	88
Pointing			
Pearson correlation	.138	.056	.124
Sig. (two‐tailed)	.190	.595	.249
Manipulating infant			
Pearson correlation	.092	−.144	.241*
Sig. (two‐tailed)	.381	.170	.023
Manipulating object			
Pearson correlation	.064	−.035	.090
Sig. (two‐tailed)	.543	.740	.407
Giving			
Pearson correlation	−.021	−.133	−.082
Sig. (two‐tailed)	.844	.205	.448
Other			
Pearson correlation	−.106	.003	.065
Sig. (two‐tailed)	.314	.979	.548
Total gesture			
Pearson correlation	.096	−.102	.196
Sig. (two‐tailed)	.362	.332	.067
Word type			
Pearson correlation	.258*	.200	.231*
Sig. (two‐tailed)	.013	.056	.030
Word token			
Pearson correlation	.321*	.137	.274**
Sig. (two‐tailed)	.002	.194	.010
Type/token ratio			
Pearson correlation	−.304**	.083	−.169
Sig. (two‐tailed)	.003	.433	.115
Total number of utterances			
Pearson correlation	.202	.034	.201
Sig. (two‐tailed)	.053	.745	.060
MLU			
Pearson correlation	.373**	.285**	.230*
Sig. (two‐tailed)	.000	.006	.031

* Denotes significant differences at *p* < .05.

** Denotes significant differences at *p* < .01.

Correlations indicate that the mean length of utterance is the most useful outcome variable for modelling the effects of socio‐economic factors in this sample, since it correlates with all three proxy measures. A hierarchical regression was run to examine the relative effects of deprivation, quality of life and maternal education on the mean length of utterance.

Model 1 revealed that SIMD accounts for 14.7% of the variation in parental MLU. The addition of WHOQOL‐BREF and maternal level of education (Model 2), led to an increase in *R* ^*2*^ of .01. This was not a statistically significant increase in model fit at the 95% confidence level *F*(2, 74) = .432, *p* = .651. The full model of SIMD, WHOQOL‐BREF and maternal level of education to predict MLU was *R* ^2^ = .157, *F*(3,74) = 4.602, *p* = .005, adjusted *R* ^2^ = .123. This model is statistically significant. SIMD had the largest effect on MLU, relative to WHOQOL‐BREF and maternal level of education (see Table 8).

**TABLE 8:** Hierarchical regression analyses reporting the relative effects of deprivation, quality of life and maternal education on mean length of utterance.

Variables	Model 1		Model 2	
	B	β	B	β
Constant	2.87		2.58	
SIMD rank	9.96E‐05	.38	8.852E‐05	.34
WHOQOL‐BREF			0.02	.06
Maternal level of education			0.03	.08
*R* ^2^	.147		.157	
*F*	13.14		4.60	
∆*R* ^2^	.15		.01	
∆*F*	.00		.65	

## DISCUSSION

Results from this study indicate a clear relationship between socio‐economic status and parental communication. Parents from a higher socio‐economic background produced a higher quantity of words, spoke with a more varied vocabulary and used longer utterances. These relations were prevalent for speech but not for gesture variables. This finding is consistent with previous research which found that mothers from a higher socio‐economic group spoke more and with a more varied vocabulary than mothers of a lower group (Hoff, 2003). We extend this work by showing that these observations from the general population also apply in a sample born preterm, a group known to experience language difficulties and for data collected in infancy.

In our data, insights varied depending on the proxy used to capture socio‐economic status. Lower neighbourhood deprivation and greater well‐being were related to larger volumes (indicated by tokens), greater complexity (as indicated by MLU) and greater variety of parental communication (indicated by types), while education related only to greater language complexity. Deprivation was predictive of mean length of utterance, but quality of life and maternal level of education scores produced only small independent effects. In our sample, neighbourhood deprivation was more influential on parental language than level of education or quality of life, and future research in a large sample could examine whether this pattern holds more generally. If the finding were found to be robust, it could point to the need for policy and public health interventions to facilitate improvements in the infant language environment. However, we also note the efficacy, at least in the short term, of individual level interventions targeting a specific family (McGillion et al., 2017) and research pointing to the importance of parent–infant interaction as an influence on language development (Der Nederlanden et al., 2025). Further research to decompose the specific deprivation factors that shape language development could help to signpost effective policy change.

Interestingly, none of our socio‐economic variables were related to the gestural output of parents. This could be because the parents were communicating with a nonverbal interlocutor, which may have limited the results. While some literature suggests socio‐economic‐related differences in the non‐verbal communication of parents, those studies draw on data from older infants, beyond their first birthday (Iverson et al., 1994; Rowe, 2000). At this point in development, infants are verbal and are beginning to form early words (Rowe & Goldin‐Meadow, 2009). It may be that parental gestures become more meaningful and specific at this stage of development, and socio‐economic differences become apparent. As our sample was only 9 months old, the way in which parents are interacting with their infants is inevitably different from how they would interact with an older child who is more advanced in terms of joint attention and early language.

The negative correlation between SIMD and type/token ratio is unusual and the opposite directionality to what the literature and other results would suggest. This may be a statistical artefact, perhaps attributable to the use of a ratio variable (Kronmal, 1993). As parents from low socio‐economic backgrounds produced fewer types and tokens compared to parents from higher socio‐economic households, small changes in type or token value can have a significant impact on the ratio. A parent who is using a larger overall volume of word tokens, would need a comparable increase in word types to achieve the same ratio. In other words, someone using few words can enlarge their type/token ratio relatively easily because the word token count is so low, whereas someone who is producing a higher volume of word tokens would have a harder time producing a proportionately high volume of word types. Future research could instead use the alternative measure of vocabulary diversity (*vocd*, Malvern & Richards, 1997) though we also note that this too is not entirely resilient to the effect of differences in size of the underlying language corpus (McCarthy & Jarvis, 2007).

### Prematurity and parental language

There was no pattern of effects between language and gesture and gestational age and birthweight, suggesting no direct effect of prematurity on parental language output. Parents of preterm, and of term‐born infants are all providing similar language exposures for their infants, in this sample measured at 9 months. However, there are other language facets which could be of future interest. Our study did not include enough fathers for an analysis based on parent gender, though we note recent literature examining this effect (Coughlan et al., 2024). We did not measure reciprocity of language and did not code for synchronicity of vocalizations with infants, for example. It could be that while parents in both groups are using similar volumes and quality of language, the timings or relationships with infant speech may be different. For example, new research indicates that preterm infants may be especially sensitive to multi‐modal language input from their parents (Doğan et al., 2025). Moreover, although the language and gesture measured in this study offer a descriptive picture of how parents are communicating verbally and nonverbally with their infant, it does not offer information as to the infant's response. Some work has highlighted differences in preterm and term‐born infants in how they perceive speech (Gonzalez‐Gomez et al., 2021) which was not captured here. Future research should examine the influence of both parental speech and gesture, and infant response, on longer term infant communication outcomes.

## CONCLUSION

Our results suggest that parental communication does not differ between parents of preterm and parents of term‐born infants. Thus, the increased risk of language delay so often cited within the preterm community may be resulting from either a biological factor, such as differences in underlying neurology, or a different environmental factor. One such candidate is socio‐economic status. It is clear from these data that deprivation is having an effect on the way in which parents communicate with their infants. In our sample, infants from higher socio‐economic status homes had parents who communicated with more words, richer vocabularies and longer utterances. There is significant reason to believe that this difference in early language environment may have serious implications for later language outcomes. Moreover, preterm children from deprived homes may be experiencing a compounded risk due to both deprivation and prematurity, which could have important implications for their language development (Ene et al., 2019).

## AUTHOR CONTRIBUTIONS

**Sinéad O'Carroll:** Conceptualization; data curation; investigation; project administration; formal analysis; writing – original draft; visualization. **Bethan Dean:** Investigation; validation; writing – review and editing. **Yu Wei Chua:** Investigation; writing – review and editing. **Victoria Ledsham:** Investigation; project administration. **James P. Boardman:** Conceptualization; funding acquisition; methodology; writing – review and editing; supervision; resources. **Sue Fletcher‐Watson:** Conceptualization; methodology; supervision; resources; writing – original draft.

## CONFLICT OF INTEREST STATEMENT

The authors declare no conflicts of interest relevant to this work.

## Data Availability

We welcome requests to access data in line with the terms and conditions, and via the data access request form, both available here: https://reproductive‐health.ed.ac.uk/theirworld‐edinburgh‐birth‐cohort‐tebc/for‐researchers/data‐access‐and‐collaboration Data are not fully open access because they cannot be anonymized (e.g. parent–child play videos) and the availability of different data types depends on whether planned analyses have taken place within the research team.
